# Whole blood transcriptomic profiles can differentiate vulnerability to chronic low back pain

**DOI:** 10.1371/journal.pone.0216539

**Published:** 2019-05-16

**Authors:** Susan G. Dorsey, Cynthia L. Renn, Mari Griffioen, Cameron B. Lassiter, Shijun Zhu, Heather Huot-Creasy, Carrie McCracken, Anup Mahurkar, Amol C. Shetty, Colleen K. Jackson-Cook, Hyungsuk Kim, Wendy A. Henderson, Leorey Saligan, Jessica Gill, Luana Colloca, Debra E. Lyon, Angela R. Starkweather

**Affiliations:** 1 Department of Pain & Translational Symptom Science and Center to Advance Chronic Pain Research, University of Maryland School of Nursing, Baltimore, Maryland, United States of America; 2 University of Delaware School of Nursing, Newark, Delaware, United States of America; 3 Graduate Program in Life Sciences Molecular Medicine, University of Maryland School of Medicine, Baltimore, Maryland, United States of America; 4 Institute for Genome Sciences, University of Maryland School of Medicine, Baltimore, Maryland, United States of America; 5 Department of Pathology Division of Molecular Diagnostics, Virginia Commonwealth University School of Medicine, Richmond, Virginia, United States of America; 6 Division of Intramural Research, National Institute of Nursing Research, National Institutes of Health, Department of Health and Human Services, Bethesda, Maryland, United States of America; 7 University of Florida Gainesville College of Nursing, Gainesville, Florida, United States of America; 8 Center for Advancement in Managing Pain, University of Connecticut School of Nursing, Storrs, Connecticut, United States of America; Universidad de Jaen, SPAIN

## Abstract

The mechanisms underlying the transition from acute to chronic pain remain unclear. Here, we sought to characterize the transcriptome associated with chronic low back pain as well as the transcriptome of the transition from acute to chronic low back pain. For the analysis, we compared the whole blood transcriptome of: (a) patients at the onset of low back pain who no longer had pain within 6 weeks after onset (acute) with patients who developed chronic low back pain at 6 months (chronic T5); and, (b) patients at the onset of low back pain (chronic T1) who developed chronic pain at 6 months with healthy pain-free (normal) controls. The majority of differentially expressed genes were protein coding. We illustrate a unique chronic low back pain transcriptome characterized by significant enrichment for known pain genes, extracellular matrix genes, and genes from the extended major histocompatibility complex (MHC) genomic locus. The transcriptome of the transition from acute to chronic low back pain was characterized by significant upregulation of antigen presentation pathway (MHC class I and II) genes and downregulation of mitochondrial genes associated with oxidative phosphorylation, suggesting a unique genomic signature of vulnerability to low back pain chronicity.

## Introduction

Low back pain (LBP) is one of the most common and costly pain conditions in the United States, and affects nearly one in ten people worldwide at any given time[1]. For most individuals with an acute LBP episode, the pain resolves within 4–6 weeks along with the ability to resume normal activities. However, an estimated 20% of individuals with acute LBP have sustained levels of pain after 4–6 weeks at a level that decreases normal activities, function, and quality of life[2,3]. Chronic LBP (cLBP), which is defined as pain in the lower back occurring for at least 3 of the past 6 months, is a common reason for initiation of long-term opioid therapy, which raises the possibility for addiction, and may be a gateway to other comorbid pain disorders[4]. Currently, cLBP is responsible for a third of work-related disability and among the ten most expensive conditions in the United States[5,6].

Heritability of LBP is estimated to range from 30–60% with a higher familial incidence being associated with increased severity of LBP[7,8]. Traditional methods for assessing vulnerability to cLBP have focused on candidate genetic polymorphisms and imaging for degenerative disc disease (DDD) or specific LBP conditions such as herniated disc, lumbar stenosis or spondylolisthesis[9–12]. However, noting that DDD is ubiquitous among adults and the degree of degeneration is not consistently associated with the presence or severity of pain, this approach has not led to a consensus list of objective risk markers[13], and consequently the pathogenesis of cLBP remains unclear.

Due to the presumed etiologic heterogeneity of non-specific cLBP, very few investigators have examined the associated molecular mechanisms even though this condition makes up approximately 90% of individuals who seek healthcare for LBP[14]. One innovative approach to studying the mechanisms leading to cLBP incorporates the perspective of systems biology and aligns with the tenets of precision healthcare that are aimed at redefining clinical diagnoses and management strategies according to the molecular signature of the condition being evaluated[15]. This approach is a significant departure from traditional clinical research based on *a priori* assumptions regarding the mechanisms underlying a specific diagnosis, and instead, seeks to identify variability in molecular pathways to refine a clinical phenotype and optimize treatment regimens[16,17].

Gene expression analysis is a key tool in biomedical research that provides rich insight into the mechanistic underpinnings of disease and defining a physiological phenotype[18]. Recent cross-sectional studies, using microarray platforms to assay the repertoire of messenger RNA (mRNA) within a tissue or cell at a specific timepoint, have identified unique mechanistic pathways in human blood cells of patients with complex regional pain disorder[19], chronic visceral pain[20], and rheumatoid arthritis[21]. However, while the transition from acute to chronic pain can be assumed to represent a spectrum, none of these studies have specifically included follow-up longitudinal approaches to enable the investigators to compare the transcriptome signatures associated with transition from acute to chronic phase transcriptome within and between participants with LBP who either develop or do not develop chronic pain.

Importantly, the acute phase of LBP represents a window of opportunity to strategically intervene; and, predicting those patients who are more prone to chronicity can help develop targeted pain therapies. Therefore, we sought to examine the cLBP transcriptome and compare it with the transcriptome during the acute phase of LBP. We chose whole blood for the analysis because it has proven to be a useful surrogate of gene expression in the peripheral and central nervous system and can be collected in a minimally invasive manner that is amenable for potential future diagnostic test development[22]. Data and samples were acquired from a completed case-control study in which participants were enrolled at the onset of acute low back pain and followed up at 6-months if they continued to have low back pain (R01NR013932, PI:Starkweather). Therefore, samples were only available at one-time point, at pain onset, for participants whose pain later resolved (acute group).

To this end, we aimed to: 1) characterize the transcriptome associated with cLBP; and, 2) characterize the transition from acute to cLBP transcriptome. To accomplish these aims, we sequenced 64 RNA samples and compared the whole blood transcriptome: (a) at onset (baseline) of low back pain in patients who no longer had pain within 6 weeks after baseline (n = 11, acute group) and at baseline in patients who developed chronic pain at 6 months (n = 13, chronic T1 group); and, (b) at 6 months after baseline in patients with chronic pain (n = 19, chronic T5 group) and healthy pain-free controls (n = 21, normal group).

## Materials and methods

### Participants

Men and women between the ages of 18–50 years of age diagnosed with an acute nonspecific LBP episode and able to read and write in English were invited to participate from primary healthcare clinics through advertisements. An acute nonspecific LBP episode was defined as pain anywhere in the region of the low back bound superiorly by the thoraco-lumbar junction and inferiorly by the lumbo-sacral junction, which had been present for >24 hours but <4 weeks duration and was preceded by at least 1 pain-free month[23]. This age range was selected to provide a more homogeneous sample in terms of general health, work status and contributing factors of persistent LBP. Recruitment and enrollment took place at two urban university health systems after approval from the Institution Review Board. Informed consent procedures were strictly followed. All participants provided written consent prior to study participation.

Patients were excluded for the following conditions: (a) pain at another site or associated with a painful condition (eg., degenerative disc disease, herniated lumbar disc, fibromyalgia, neuropathy, rheumatoid arthritis, sciatica); (b) previous spinal surgery; (c) presence of neurological deficits; (d) history of comorbidities that affect sensorimotor function (e.g., multiple sclerosis, spinal cord injury, diabetes); (e) pregnant or within 3-months postpartum; (f) taking opioid, antidepressants or anticonvulsant medication; and, (g) history of psychological disorders (major depression, bipolar disorder, schizophrenia) because of a possible associations with biological markers[24–26]. Eligibility for the healthy no-pain control group included men and women (a) between 18–50 years of age; (b) could read and write in English; (c) with no known medical, psychological problems or prescribed medication; (d) not pregnant or breastfeeding; and, (e) no recent history of pain at any location.

### Procedures

After obtaining written consent, participants were scheduled to undergo baseline data collection as soon as possible but no longer than one week from the time of consent. Data collection took place in a private research suite to complete questions about age, gender, socioeconomic status, educational attainment, lifestyle behaviors (smoking, exercise), comorbidities, and past episodes of LBP. Following completion of the questionnaires, participants underwent venipuncture for collection of blood samples and quantitative sensory testing (QST). The same sequence of data collection was followed for all participants. Data collection visits were repeated at 6 months only if the participant continued to report pain.

### Study measures

#### Demographics

Age, gender, socioeconomic status, educational attainment, lifestyle behaviors (smoking, exercise), body mass index, comorbidities, and past episodes of LBP were collected at baseline.

#### Perceived pain

The Brief Pain Inventory-Short Form (BPI-SF) is a pain assessment tool that has well-established reliability and validity for adult patients with persistent pain[27] and is sensitive to change over time[28]. The BPI assesses the severity of pain, location of pain, pain medications, amount of pain relief in the past 24 hours and the past week, and the impact of pain on daily functions[28]. To measure the affective and sensory descriptors of pain, the McGill Pain Questionnaire Short-Form (MPQ-SF) was also used. The MPQ-SF is a reliable self-report measure of pain perception[29,30]. It entails 15 verbal descriptors of sensory and affective dimensions of pain and is scored on a 4-point scale (0-none to 3-severe) by adding the numeric value of each pain dimension. Higher scores indicate higher levels of sensory and affective components of pain (range 0–45).

#### Quantitative sensory testing

Quantitative sensory testing (QST) was used to evaluate responses to experimental pain elicited with standardized stimuli to test both nociceptive and non-nociceptive systems[31]. Quantitative sensory testing was performed in the lumbar region (at the location of pain) and on the dominant forearm (remote area). A standardized protocol of administration, including examination room conditions and instructions provided for the participant, were strictly followed. Participants were given a practice run on the non-dominant forearm in order to verify the participant's understanding of the protocol.

Mechanical pain threshold and sensitivity were measured with a standard set of von Frey hairs (Optihair_2_-Set, Marstock Nervtest, Germany) that exert forces between 0.25 and 512 mN with a rounded tip that is 0.5 mm in diameter. The final threshold is calculated as the geometric mean of five series of ascending and descending stimuli intensities. Wind-up ratio (WUR) was determined from this series with the mean pain rating of trains divided by the mean pain rating to a single stimuli. Dynamic mechanical allodynia (ALL) was tested using a standardized brush applied five times with a single stroke; the pain rating to each stroke was recorded.

Thermal and pressure testing was performed using the Medoc Pathway System (Ramat Yishai, Israel). The Medoc thermode, with contact area of 7.84 cm^2^, was placed in contact with the participant's skin in the area to be tested. The Medoc software guided the examiner through a series of thermal testing procedures in the following order: cold detection threshold, warm detection threshold, cold pain threshold, and heat pain threshold. The mean threshold temperature of three consecutive measurements were calculated and used for analysis. All thresholds were obtained with ramped stimuli (1°C/second) that were terminated when the participant pressed a button attached to the Medoc device. Cut-off temperatures were 0° and 50° C with a baseline temperature of 32°C. For pressure pain threshold, the examiner used an algometer (range from 50–600 kPa) attached to the Medoc Pathway system to increase the pressure at a steady rate (30 kPa/s) until the participant indicated first pain sensation by pressing the button. The pressure pain threshold (PPT) was determined by repeating the procedure at the same site until either: (1) Two values were recorded within 20 kPa of one another or (2) Three trials were administered. In either case, the mean of the two closest values were recorded as the threshold estimate. During the testing, the computer screen was positioned so that the participant was not able to watch temperature and pressure fluctuations.

#### Blood collection and processing

Blood samples collected in PAXgene tubes were centrifuged and stored at -80°C for bulk processing following the manufacturer’s protocol. RNA isolation was performed using the PAXgene total RNA isolation system (Qiagen, Valencia, CA) according to the manufacturer’s protocol and was reverse transcribed using iScript cDNA synthesis kit (Invitrogen, Valencia, CA). Extracted RNA was run on Bioanalyzer gels to obtain the RNA integrity number (RIN; those used were at least 8/10 on the quality score).

#### RNA-seq methods

RNA sequencing was performed on all of the samples using the Illumina HiSeq sequencing technology following the manufacturer’s protocol. Raw sequences at a read length of 151 base pairs (bp) were obtained in FastQ format files. On average, 86 million paired reads per sample were generated. Read quality was assessed using the FastQC toolkit[32] to ensure good quality reads were used for downstream analyses.

#### RNA-seq analysis

The raw sequencing reads were used as input for the TopHat alignment tool (v2.1.1)[33]. TopHat is a splice-aware aligner for RNA-Seq reads which aligns reads to mammalian sized reference genomes. The reads were aligned to the human reference genome (GRCh38) downloaded from Ensembl[34] allowing for maximum 2 mismatches in each 25 bp segment and a maximum of 20 alignment hits per read. The alignment results were sorted and indexed for downstream analyses as BAM format files. The aligned reads were further utilized to generate gene expression counts using the HTSeq count tool[35] against the human reference annotation (GRCh38.86) which generates raw read counts for each genetic feature using the uniquely mapped reads. The raw read counts for each gene were further normalized for library size and gene length to generate ‘Reads per kilobase of the gene per million mapped reads (RPKM)’. The gene expression values were further utilized to assess differential expression between phenotypic conditions using R package ‘*DESeq*’[36]. DESeq provides methods to test for differential expression by use of the negative binomial distribution and a shrinkage estimator for the distribution’s variance. P-values are generated using a modified Fisher’s exact test provided within DESeq and further corrected for multiple hypothesis testing using the Benjamini-Hochberg correction method to decrease the false discovery rate (FDR). Significant differential expressed genes were yielded at a false discovery rate (FDR) of 5% and a minimum fold-change of 1.5X. Additional functional analyses were generated through the use of IPA (https://www.qiagenbioinformatics.com/products/ingenuity-pathway-analysis).

#### Validation qPCR

Custom primer sets were developed by the Institute for Genome Sciences, University of Maryland, Baltimore, for validation qPCR. For analyses, delta Cq values for each gene were normalized to the average expression of that gene in the pain-free healthy control group. Results are based on normalization using the average of the three most stable HKGs (*GAPDH*, *ACTB*, and *B2M*). For each of the non-housekeeping genes considered “genes of interest” (GOI), the delta Cq value was calculated using the quantification cycle value method to determine the normalized GOI expression level fold change. For each comparison, a Univariate Analysis of Variance (ANOVA) was used to compare the fold change between groups.

#### Statistical analysis

Due to the design of the study and lack of repeated data collection in the acute and normal groups, a time series analysis (repeated measures analysis) was not possible. Therefore, we compared data from 11 participants at the onset of low back pain who were pain-free by 6 weeks (acute) and at the onset of low back pain in the chronic group (chronic T1; six of the baseline samples did not meet quality control standards and were not sequenced for a total of 13 samples). We also compared data from 19 participants who continued to have pain at 6 months (chronic T5) and 21 pain-free normal controls. Normality of the data were tested using the Kolmogorov-Smirnov test. Student t-tests were used to test for group differences in demographic and QST variables that were normally distributed, whereas the Kruskal-Wallis test followed by Bonferroni-Holm adjusted Mann-Whitney *U* test for post hoc analyses were used for variables that were not normally distributed. Categorical variables were compared using **χ** ^2^ tests. Post-hoc analyses were conducted as necessary to account for multiple testing. For Euclidean clustering, model-based clustering for RNA-seq data was performed in R statistics as described by Si et al[37].

## Results

### Participant demographics

Across the 4 groups (normal, acute, chronic T1, chronic T5) there were no significant differences in age or gender, however the groups were significantly different with respect to race, with a greater number of White participants in the normal control group compared to the acute and chronic T1 groups; more Black participants in the chronic T5 group compared to the normal control and acute groups; and more participants in the “other” racial category compared to the acute, chronic T1 and chronic T5 groups (Table 1). No significant differences were found between groups for body mass index (BMI), number of exercise days, hours of sleep, or general medication use (S1 Table). However, the acute group was significantly more likely to be taking pain medication (nonsteroidal anti-inflammatory drugs/acetaminophen) than normal controls (Table 1). The chronic group was significantly less educated, and underemployed compared to the normal and acute group (S1 Table).

**Table 1:** Demographics and pain phenotype.

	Normal (healthy controls) N = 21	Acute N = 11	Chronic-baseline (T1) N = 13	Chronic- 6 months (T5) N = 19	P-value
Age, mean (SD)	30.9 (13.9)	34.4 (9.1)	38.5 (8.4)	38.7 (9.0)	0.184
Gender					0.993
Male (%)	9 (42.9)	5 (45.5)	6 (46.2)	9 (47.4)	
Female (%)	12 (57.1)	6 (54.4)	7 (53.8)	10 (52.6)	
Race					<0.001
White (%)	12 (57.1)	6 (56.0)	6 (46.2)	8 (42.1)	
Black (%)	1 (4.8)	3 (27.0)	7 (53.8)	11 (57.9)	
Other (%)	8 (38.1)	2 (18.0)	0 (0.0)	0 (0.0)	
Pain score right now (0–10) mean (SD)	0 (0.0)	2.9 (0.9)	5.5 (2.5)	5.7 (2.8)	<0.001
Pain average score (0–10) mean (SD)	0 (0.0)	3.4 (1.6)	5.2 (2.0)	5.1 (2.4)	<0.001
Heat pain tolerance mean (SD)	43.3 (3.7)	40.5 (4.6)	40.0 (3.0)	39.5 (3.3)	0.008
Pain medication (opioid) mean (0 = no, 1 = yes) (SD)	0 (0.0)	0 (0.0)	0 (0.0)	0 (0.0)	—
Pain medication (NSAID/Tylenol) mean (0 = no, 1 = yes) (SD)	0 (0.0)	0.64 (0.50)	0.23 (0.44)	0.32 (0.48)	<0.001

^a^p-values were calculated from ANOVA or Chi-square test(s), whenever applicable

### Characterization of the chronic LBP transcriptome

**Fig 1:**
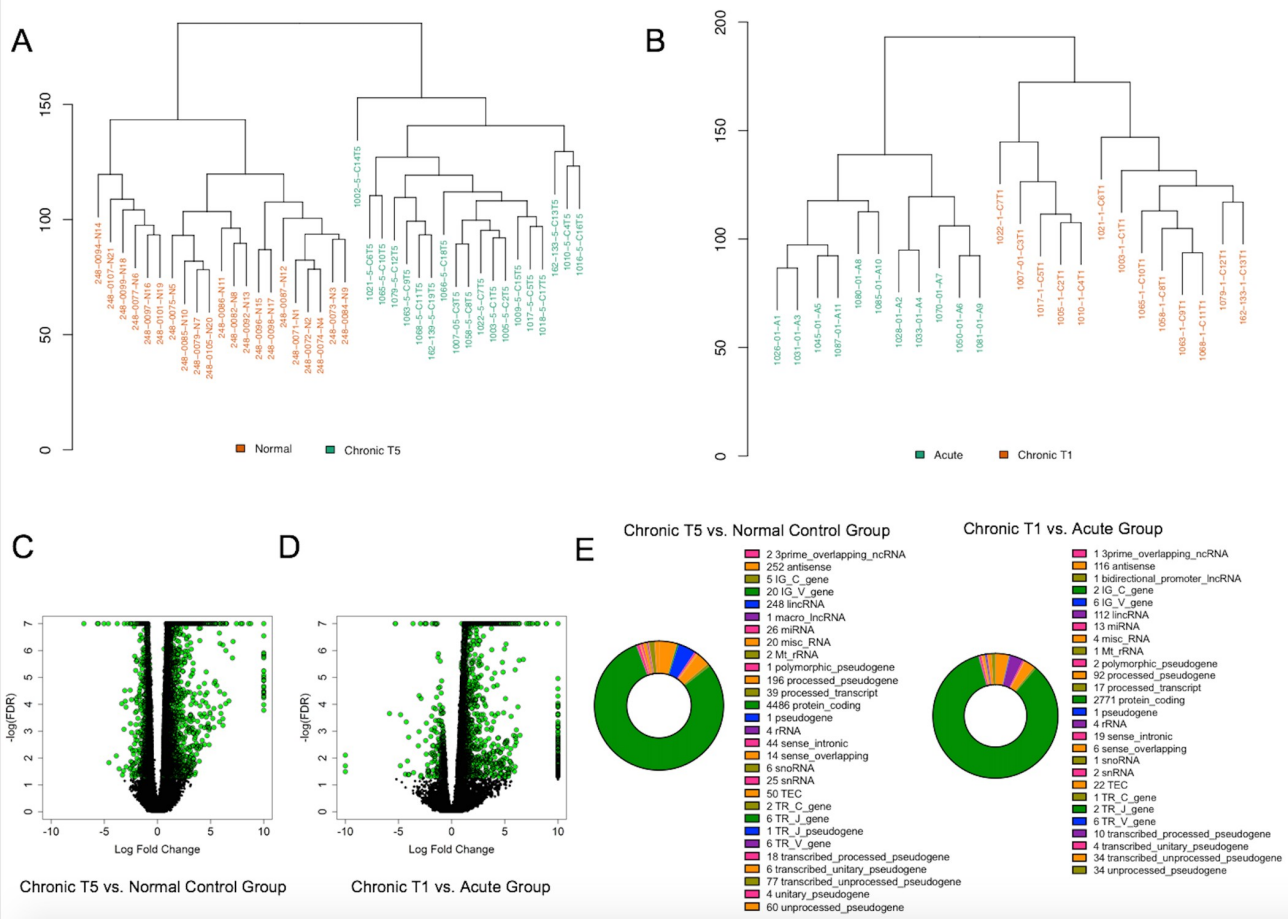
Characterization of differential gene expression in whole blood from the chronic T5 versus normal (healthy pain-free) group and in the chronic T1 and acute group. (A) Euclidean clustering demonstrates separation between the chronic T5 group compared with the normal control group. (B) Euclidean clustering demonstrates separation between the acute and chronic T1 group. (C) Volcano plot showing log fold change (LFC) for significantly differentially expressed genes (LFC ±0.58; FDR p-value ≤ 0.05; N = 3,479 shown in green circles) in the chronic T5 versus normal group. (D) Volcano plot showing log fold change (LFC) for significantly differentially expressed genes (LFC ±0.58; FDR p-value ≤ 0.05; N = 3,288 shown in green circles) in the chronic T1 compared with the acute group. (E) Pie charts depicting protein-coding and non-coding gene types for chronic T5 versus normal group (left) and chronic T1 versus acute group (right).

We obtained an average of 86.7 million 150 base pair (bp) paired end reads per sample. Of those, an average of 81.9% of the read pairs mapped to the reference genome (Ensembl Homo_sapiens GRCh.38.p.7 release 86) and 80% were properly paired. The majority were exonic (90.3%), with 7.1% on average intronic and 2.6% intergenic. This enabled us to achieve an average of 32X coverage of the transcriptome. We examined differential expression of aligned reads using DESeq, and a gene was identified as differentially expressed if it showed a log fold change (LFC) ±0.58 and had a False Discovery Rate (FDR) p-value ≤ 0.05 based on previous criteria established in assessing differentially expressed genes in pain populations[22]. We performed Euclidean clustering on the differentially expressed gene (DEG) set of 5,632 genes and found that the chronic T5 group was distinctly clustered from the normal (healthy pain-free) group, and that the acute group distinctly clustered from the baseline chronic T1 group (Fig 1). There were 3,479 significantly differentially expressed genes between the chronic T5 group and normal controls and 3,288 significantly differentially expressed genes between the acute and chronic T1 group.

**Fig 2:**
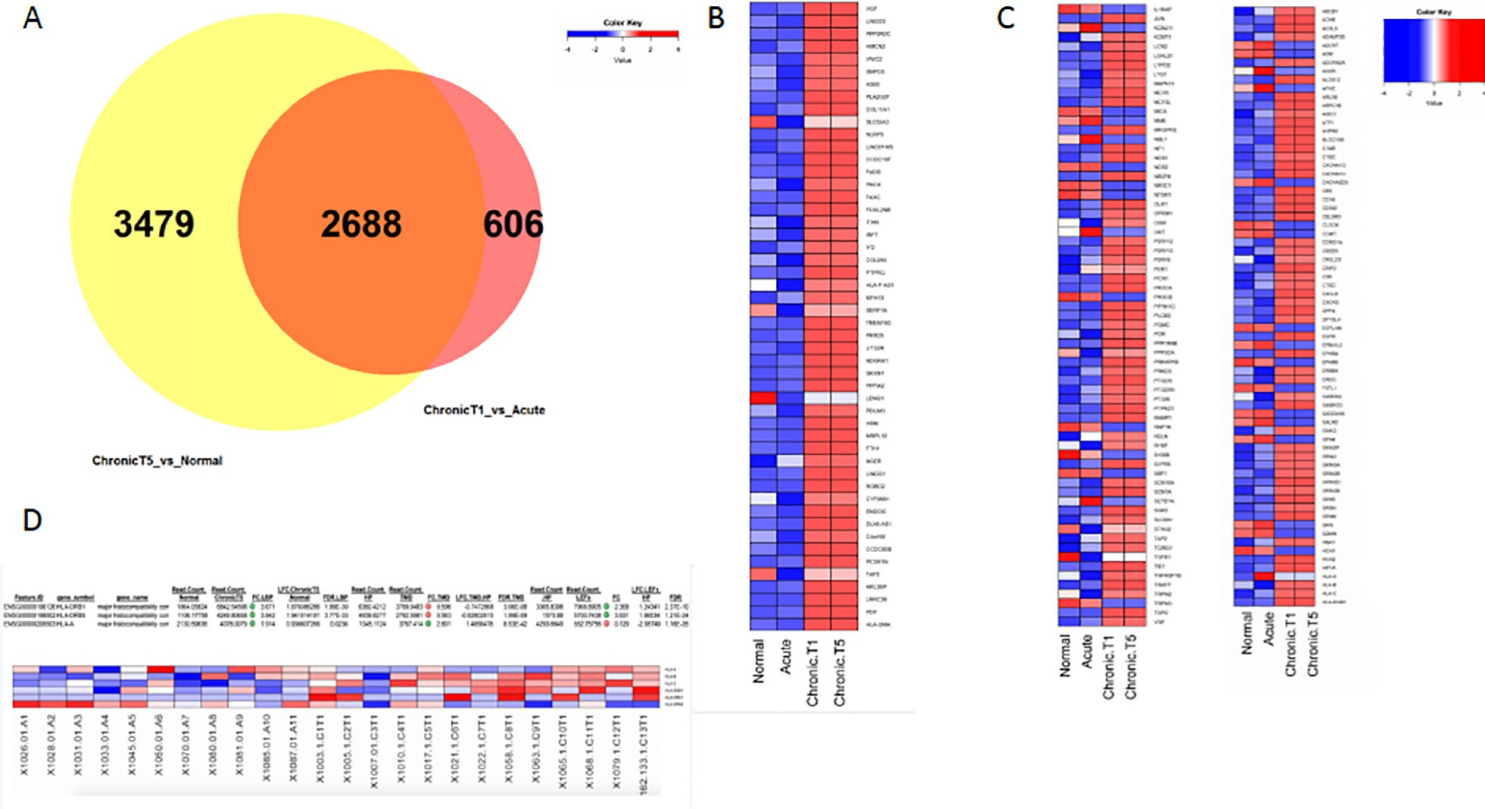
Comparisons in differential gene expression among normal control (single timepoint) and cases (acute and chronic T1 and T5). (A)Venn diagram showing that 2,688 differentially expressed genes (LFC ±0.58; FDR p-value ≤ 0.05) overlap between the two contrasts. (B) Heat map showing the top 50 up-regulated (left) and down-regulated (right) protein-coding genes in all four groups [healthy participants (normal), acute, chronic T1 and chronic T5]. Color key shows the z-score for downregulated genes (blue) and upregulated genes (red). (C) A one-tailed Fisher’s Exact test was used to compute a hypergeometric p-value to determine whether the differentially expressed genes from each contrast (chronic T5 versus normal control group and chronic T1 versus acute group) were significantly enriched for known pain genes (dataset constructed from multiple literature and online database sources (see supplemental methods for detail). The p-value for known pain genes = 1.26E-09 for the chronic T5 versus normal participants contrast, and p = 2.62E-08 for the chronic T1 versus acute contrast and for genes that form the extended MHC locus. The heatmap depicts known pain genes. Color key shows the z-score for downregulated genes (blue) and upregulated genes (red). (D) We computed the one-tailed Fisher’s Exact test to obtain a p-value for genes that reside in the extended MHC genomic locus (see supplemental methods for detail). The p-value for genes in the extended MHC genomic locus was only significant for the chronic T1 versus acute contrast (p = 1.43E-02).

The majority of DEGs were identified as protein coding (Fig 1). There were 2,688 significantly differentially expressed genes between the contrasted groups (Fig 2). The top 100 DEGs in the chronic T5 compared with the normal group were identified and plotted in context with the acute and chronic T1 data (Fig 2). The DEG set that differentiated the chronic T5 group from the normal group was significantly enriched for known pain genes (Fig 2C; complete list can be found in S2 Table) and genes on the extended major histocompatibility complex (MHC) locus (Fig 2).

**Fig 3:**
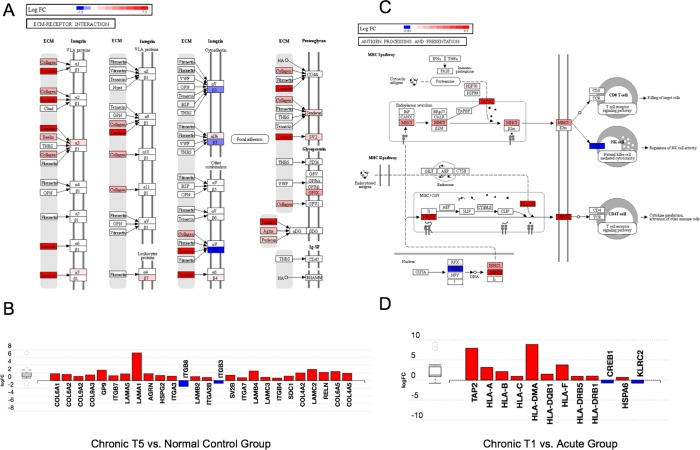
Unbiased pathway analysis demonstrates significant enrichment for extracellular matrix genes in chronic T5 versus the normal control group and the antigen presentation pathway (MHC class I and II) genes in the chronic T1 patients compared with the acute group. Using the Impact Analysis method in iPathway (Advaita Corporation), we conducted unbiased pathway analysis. Each pathway diagram is overlayed with the computed perturbation of each gene. The perturbation accounts both for the gene's measured fold change and for the accumulated perturbation propagated from any upstream genes (accumulation). The highest negative perturbation is shown in dark blue, while the highest positive perturbation is shown in dark red. The legend describes the values on the gradient in logFC. Note: For legibility, one gene may be represented in multiple places in the diagram and one box may represent multiple genes in the same gene family. A gene is highlighted in all locations it occurs in the diagram. For each gene family, the color corresponding to the gene with the highest absolute perturbation is displayed. (A) Top differentially regulated pathway in the chronic T5 versus normal group is extracellular matrix (ECM)-receptor interaction (KEGG: 04512; p = 0.005). (B) Bar graph of individual gene display for the ECM-receptor interaction pathway. The signed perturbation is represented with negative values in blue and positive values in red. The box and whisker plot on the left summarizes the distribution of all gene perturbations in this pathway. The box represents the 1st quartile, the median and the 3rd quartile, while circles represent the outliers. (C) Top differentially regulated pathway in the chronic T1 versus acute group is antigen processing and presentation (KEGG: 04612; p = 0.006). (D) Bar graph individual gene display for the antigen processing and presentation pathway. The signed perturbation is represented with negative values in blue and positive values in red. The box and whisker plot on the left summarizes the distribution of all gene perturbations in this pathway. The box represents the 1st quartile, the median and the 3rd quartile, while circles represent the outliers.

To assess replication of RNA seq results from selected targets in each of these datasets we performed qPCR (primer sets in S3 Table). Upregulated expression of the *HLA-A*, *HLADRB5*, *PDF* genes from RNA seq and qPCR were consistent (S1 Fig). We next asked what biological pathways were enriched in the DEG set between normal controls and chronic T5 group, and the top pathway identified was the extracellular matrix (ECM) receptor interaction including collagen genes, laminin genes, and others (Fig 3). The biological pathway enriched in the DEG set between chronic T1 and acute group was the antigen processing and presentation pathway including many HLA genes (Fig 3).

**Fig 4:**
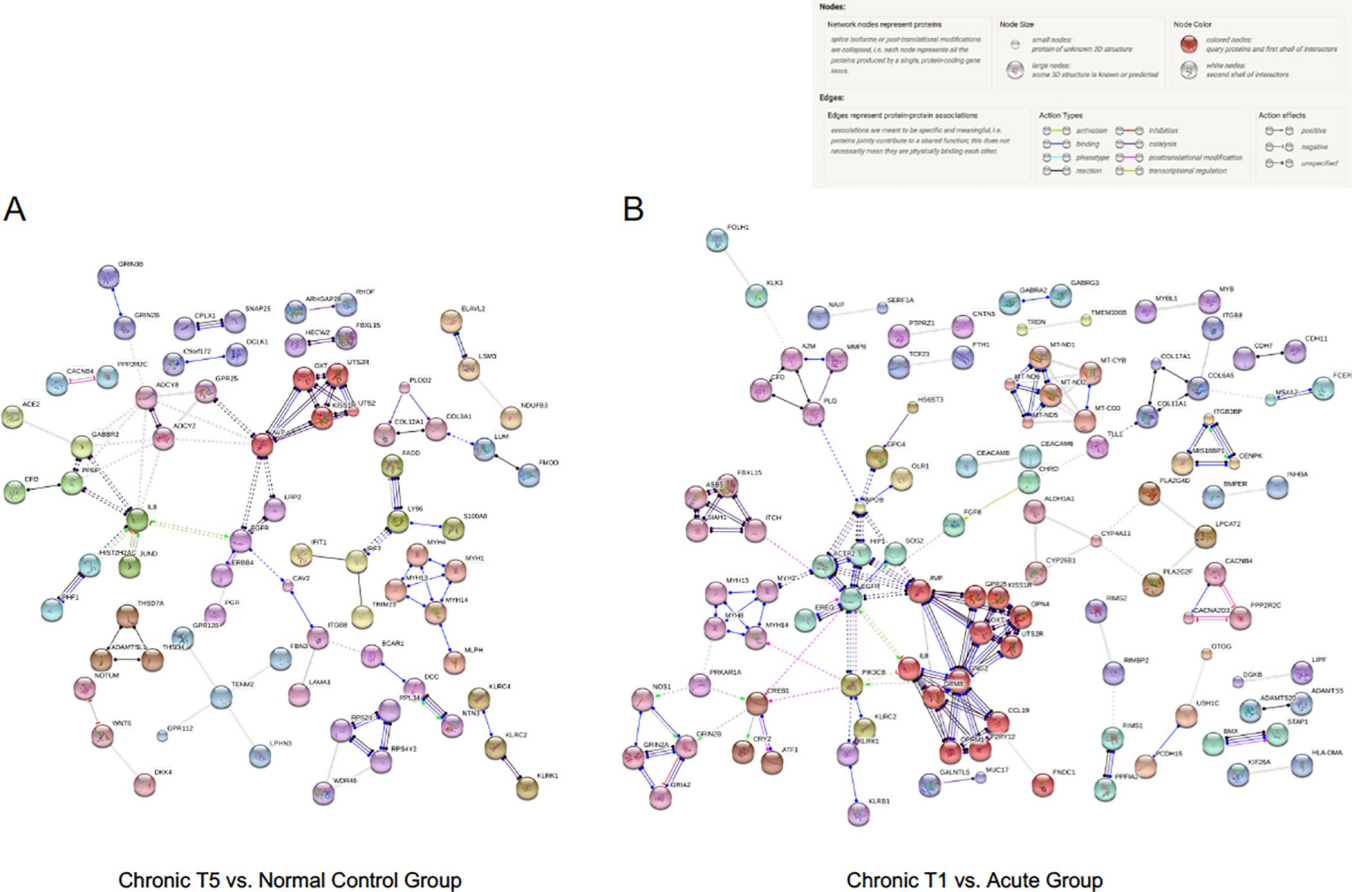
Protein-protein interaction network analysis demonstrates clusters of co-expressed genes. Evidence for significant protein-protein interactions was demonstrated using STRING v10. For both contrasts (chronic T5 versus normal controls; chronic T1 versus acute), we analyzed the top 500 differentially expressed genes (250 upregulated, 250 downregulated). For the analysis, we specified high confidence (0.70) for cluster positions in the network as determined by an algorithm that computes a global confidence binding score. We next removed all disconnected nodes and then applied the Markov Cluster Algorithm (MCA) to extract clusters of densely connected nodes from biological networks. (A) String v10 analysis of gene expression analysis from chronic T5 compared with normal controls. Of the top 500 differentially expressed genes, 327 were identified in the database. The final network is comprised of 327 nodes and 150 edges. The random number of edges is 92. The protein-protein interaction (PPI) enrichment p-value = 1.88E-08. (B) String v10 analysis of gene expression in chronic T1 compared with the acute group. Of the top 500 differentially expressed genes, 263 were identified in the database. The final network is comprised of 263 nodes and 84 edges. The expected number of edges is 64. The PPI enrichment p-value = 0.0107.

**Fig 5:**
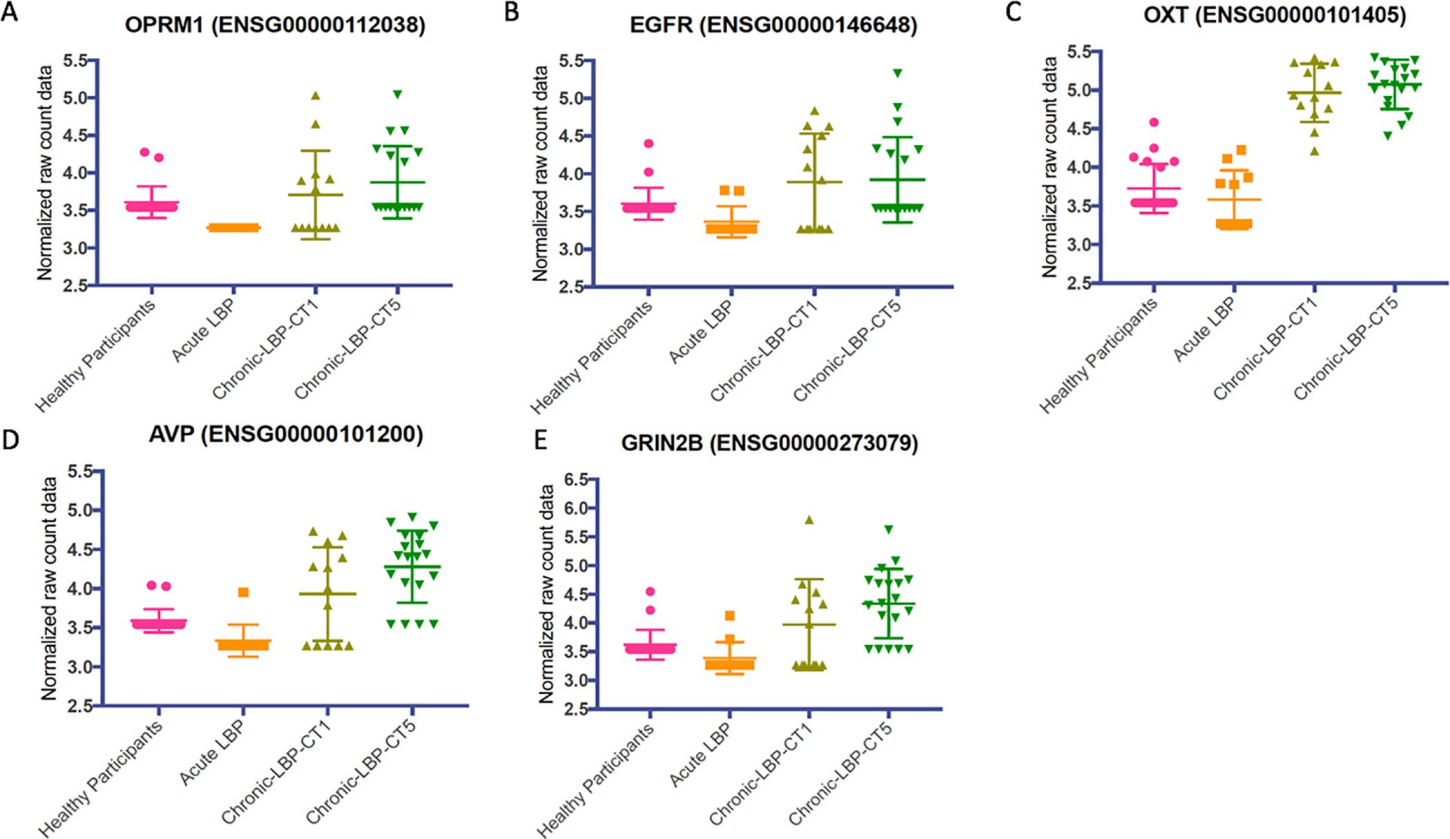
The whole blood gene expression levels for known or suspected pain genes that were members of major clusters from String 10 protein-protein interaction analysis were plotted. The analysis demonstrates that levels of each gene are differentially higher in the chronic T1/chronic T5 patients compared with acute and normal control participants. In each panel, the raw, normalized, non-zero gene expression counts from DESeq analysis are displayed as a scatter plot for each cohort. Symbols indicate individual expression levels for each of the N = 64 participants. A Kruskal-Wallis 1-way ANOVA was used to examine comparisons. In all cases, the expression levels for each gene were higher in the chronic T1 and chronic T5 groups compared with normal control participants and the acute group. (A) Opioid receptor mu 1 (*OPRM1*) gene (H = 27.64, p< 0.0001). (B) Epidermal growth factor receptor (*EGFR*) gene (H = 15.29, p = 0.0016). (C) Oxytocin (*OXT*) gene (H = 47.43, p<0.0001). (D) Arginine vasopressin (*AVP*) gene (H = 31.44, p<0.0001). (E) Glutamate ionotropic receptor NMDA type subunit 2B (*GRIN2B*) gene (H = 24.25, p<0.0001).

Co-expression analysis of the DEG set using STRING v10 showed several gene clusters, including *OXT*, *EGFR* and *TEMN2* (Fig 4). We then compared the expression levels (normalized read counts) at the gene level for selected genes from the co-expression analysis, and found that while some participants had levels of each gene that were comparable with normal participants, many of the chronic T5 patients had statistically higher levels of each gene (Fig 5).

**Fig 6:**
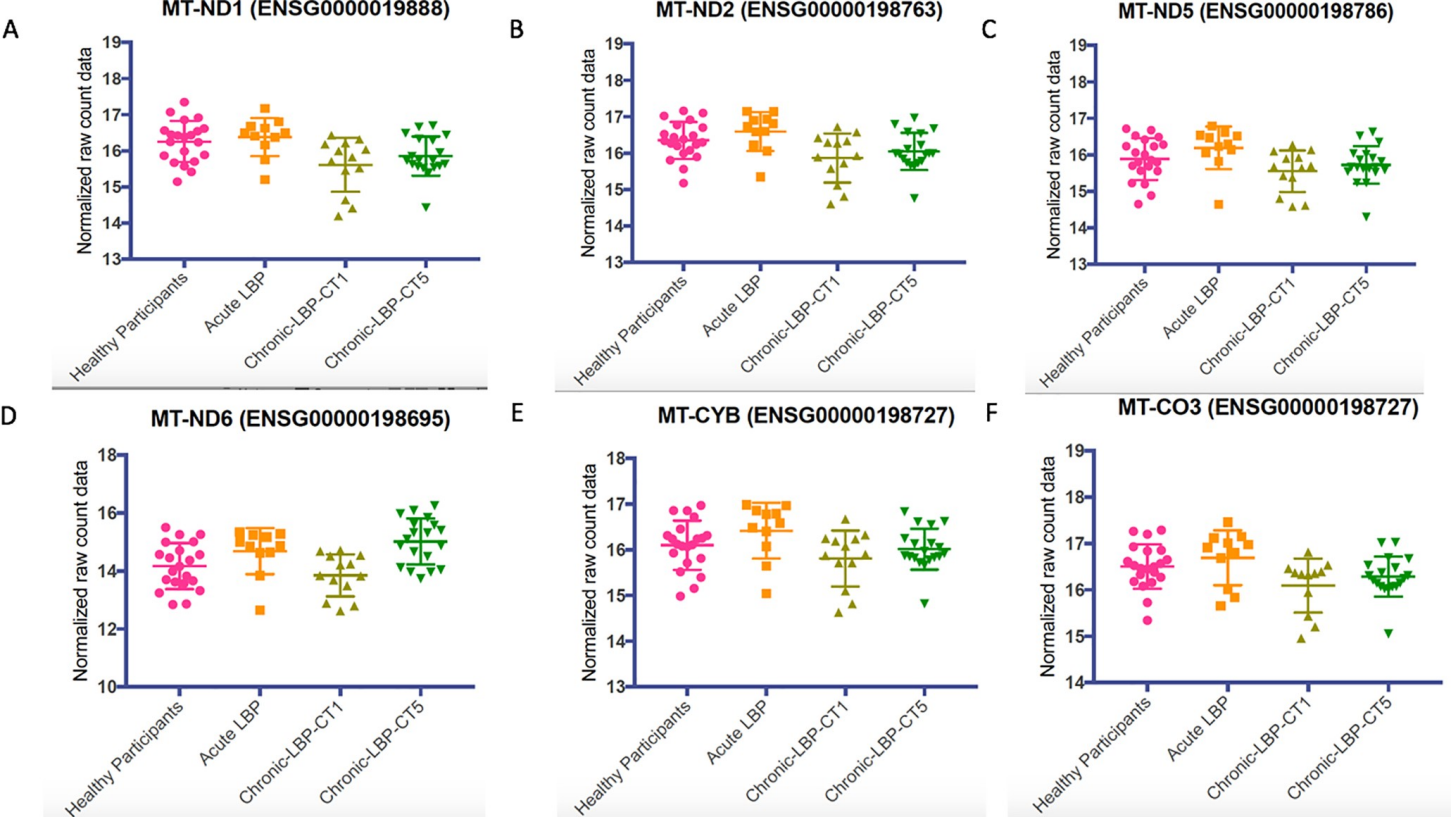
The whole blood gene expression levels for a cluster of mitochondrial genes associated with oxidative phosphorylation from String 10 protein-protein interaction analysis were plotted. Results demonstrate that levels of each gene are differentially lower in the chronic T1 compared with the acute and normal participants. In each panel, the raw, normalized, non-zero gene expression counts from DESeq analysis are displayed as a scatter plot for each cohort. Symbols indicate individual expression levels for each of the N = 64 samples. A Kruskal-Wallis 1-way ANOVA was used to examine comparisons. In all cases except *MT-CYB*, the expression levels were statistically significantly lower in chronic T1 patients. (A) Mitochondrial encoded NADH dehydrogenase 1 (*MT-ND1*) gene (H = 12.29, p = 0.0064. (B) NADH dehydrogenase 2 (*MT-ND2*) gene (H = 12.05, p = 0.0072). (C) NADH dehydrogenase 5 (*MT-ND5*) gene (H = 10.22, p = 0.0168). (D) NADH dehydrogenase 6 (*MT-ND6*) gene (H = 17.03, p = 0.0007). (E) Cytochrome B (*MT-CYB*) gene (H = 7.333, p = 0.0620). (F) Cytochrome C oxidase III (*MT-CO3*) gene (H = 8.146, p = 0.0431).

We next examined the whole blood gene expression levels for a cluster of mitochondrial genes associated with oxidative phosphorylation from the String 10 protein-protein interaction analysis (Fig 4). This was performed for the 64 samples obtained from normal controls, acute, chronic T1 and chronic T5. In all cases except *MT-CYB*, the expression levels were statistically significantly lower in chronic T1 patients (Fig 6).

### Characterization of the transition from acute to chronic LBP transcriptome

**Fig 7:**
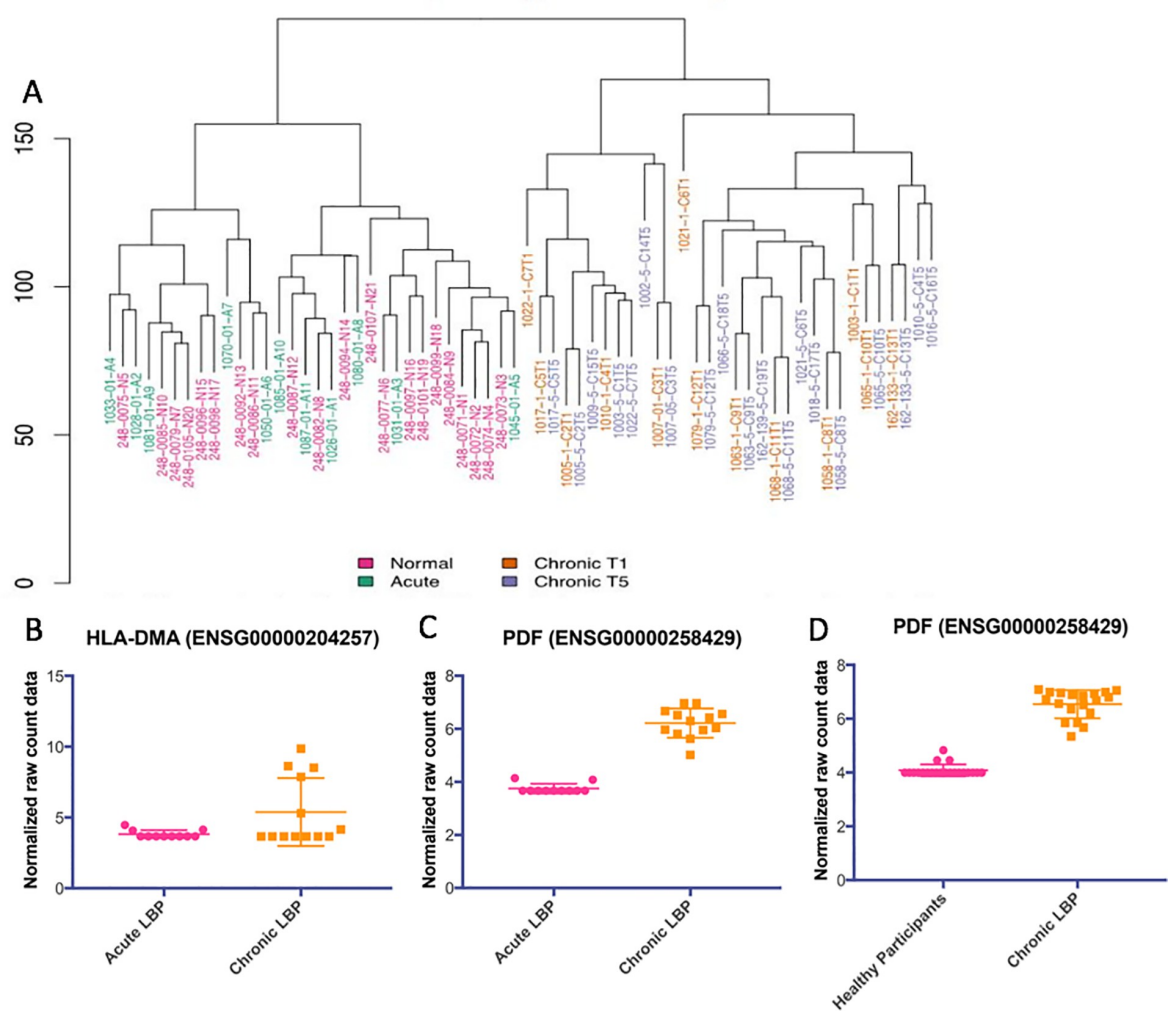
Characterization of differential gene expression in whole blood over the transition from acute to chronic low back pain. (A) Euclidean clustering demonstrates separation between normal control/acute group and chronic T1/T5. (B and C) *HLA-DMA* and *PDF* were the top 2 differentially expressed genes between the acute group and chronic T1 group. (D) *PDF* was significantly differentially expressed between normal controls and the chronic T5 group.

To characterize the transition from acute to chronic pain, we performed Euclidean clustering on the set of differentially expressed genes (DEG) and found that the chronic T1/T5 patients were distinctly clustered from the normal control and acute patients (Fig 7). The top 100 DEGs in the chronic patients (CT1 and CT5) were identified and plotted in context with the normal controls and acute group (Fig 2). In examining the heatmaps in Fig 2, it was apparent that the normal control and acute group transcriptomes were more similar than the two chronic T1/T5 patient transcriptomes. The top two differentiated genes between acute and chronic T1 groups were *HLA-DMA* and *PDF* (Fig 7). Mitochondrial gene, Peptide deformylase *(PDF)*, was significantly upregulated at T1—baseline compared to the acute group and at T5–6 months compared to the normal control group (Fig 7). We next compared selected targets from the RNA seq analysis of the chronic T5 transcriptome using qPCR to confirm upregulated expression in the *HLA-A*, *HLADRB5*, and *PDF* genes (S1 Fig). Finally, we conducted unbiased gene ontology analyses, and the most significant pathway was the antigen processing and presentation pathway (Fig 3), demonstrating that higher levels of HLA and MHC locus genes were characteristic of the chronic T1 patients versus acute patients. Co-expression analyses of the DEG list showed several main clusters of genes centered on *OPRM1*, *EGFR*, *OXT*, *AVP*, and *GRIN2B* (Fig 5).

## Discussion

This study took an innovative approach of following patients prospectively at the onset of acute low back pain to monitor the transition to resolution or chronicity of low back pain and analyzing the whole blood transcriptome of this process. We identified a unique cLBP transcriptome characterized by significantly differentially expressed genes enriched for known pain genes, with significant upregulation of ECM genes, and genes from the extended MHC genomic locus. The chronic T1 and T5 groups demonstrated significant upregulation of *OPRM1*, *EGFR*, *OXT*, *AVP*, and *GRIN2B*, which are genes known to be involved in pain processing. In respect to the acute to chronic transcriptome, we identified significant downregulation of mitochondrial genes involved in oxidative phosphorylation, *MT-ND1*, *MT-ND2*, *MT-ND5*, *MT-ND6*, and *MT-CO3* in the chronic T1 group compared to the normal and acute groups. We also demonstrate that, at the gene level, the transition from acute to cLBP is characterized by upregulation of HLA and MHC locus genes, including, *HLA-DMA* and *PDF*, with continued upregulation of *PDF* at 6-months from the onset of pain.

### Characterization of the transcriptome associated with chronic low back pain

Similar to other cohorts of non-specific, or axial LBP, we identified several social determinants of health that differed between the acute and cLBP groups, including levels of education and employment. However, patients in this sample did not differ on other socio-economic factors, body mass index or daily physical activity. Using Euclidean cluster analysis of our gene expression data described in the methods (see section Euclidean Clustering), we found that the sample separated into two distinct groups, healthy controls/acute versus chronic T1/T5, based solely on their gene expression profiles.

Further, the performed analyses revealed that the list of genes differentially expressed between the groups is highly enriched for genes known to be associated with pain, including the extended MHC locus. This finding is indeed in line with the dearth of literature demonstrating that the MHC locus has been found to be enriched in DRG tissue from a post herpetic pain model in mice[38]. Moreover, the MHC complex was also shown to play a role in pain behavior in a neuropathic pain model[39–41] in an MHC complex congenic strain of rats. Investigators have also examined the role of MHC gene expression in humans with chronic pain. MHC genes were found to be upregulated in patients with herpes simplex virus-induced unilateral neuropathic-like pain[42], Reiter’s disease, a chronic condition that includes low back pain and stiffness as a symptom[43] and a cross-sectional study of patients with temporomandibular disorder or low back pain[22]. Noting that the control sample of the current study had less Black participants compared to the chronic T5 group, one does need to be cautious in drawing conclusions about DEGs, such as those in the MHC region, that show different patterns in racially diverse groups. This finding requires further studies in larger samples with adequate power to control for sex and race/ethnicity or that include matched comparison groups, which is a major foci of this team of investigators. Nevertheless, it is known that the human leukocyte antigen (HLA) locus of the MHC region is frequently associated with various chronic pain phenotypes, and was found to have representative genes upregulated in this study. We found that all three of the MHC class I genes *(HLA-A*, *HLA-B*, *HLA-C*) and three of the six MHC class II genes (*HLA-DQB1*, *HLA-DRB1*, and *HLA-DRB5*) were upregulated in the chronic T1 compared to the acute group. MHC class I gene upregulation has been associated with chronic regional pain syndrome (CRPS)[44–46], chronic pancreatitis[47], and post herpetic neuralgia[48,49]. MHC class II gene upregulation has also been associated with chronic pain conditions including chronic pancreatitis[49–50], inguinal hernia repair[51], CRPS[19], lumbar disc herniation[51], rheumatoid arthritis[52,53], chronic inflammatory response syndrome (CIRS), which includes pain as a major symptom[54] and in TMD and LBP[22]. Taken together, the findings from the cLBP transcriptome analysis compared to healthy normal controls in this study are in line with what is found in the literature and suggests that upregulation of the genes found in the MHC locus might form a transcriptomic signature of patients with cLBP.

### Characterization of the acute to chronic pain transcriptome

However, the analysis above does not answer the question of what genes are differentially expressed between LBP patients whose pain resolved and those who developed chronic LBP. Thus, we compared the baseline transcriptomic profiles (at pain onset) of patients whose pain resolved within six weeks (acute group) with those who developed cLBP (chronic T1 and T5). This analysis was important to determine whether there was a genetic difference between the groups as well as an expression profile that indicates increased risk of progressing to cLBP. As with the chronic T5 versus healthy controls, Euclidean cluster analysis of our gene expression data showed that the sample separated into two distinct groups, acute versus chronic T1, based solely on their transcriptomic profile. Further, like the previous analysis, the list of genes differentially expressed between the groups was highly enriched for known pain genes and genes in the extended MHC locus. This is an important finding because, unlike the initial analysis that described the cLBP transcriptomic signature in the chronic pain phase, these genes could potentially represent whole blood biomarkers to predict which LBP patients are at a higher risk for developing cLBP. Genes in the MHC locus have been associated with nocifensive behaviors in preclinical studies of noxious acute pain stimuli[55,56] in an MHC complex congenic strain of rats and complete Freund’s adjuvant inflammation in MHC II mutant mice[22].

Finally, we performed an unbiased pathway analysis of the differentially expressed genes from the previous comparisons (chronic T5 vs. normal controls and acute vs. chronic T1). In the chronic T5 group versus normal controls comparison, there was significant enrichment for extracellular matrix (ECM) genes. The top differentially regulated pathway was the ECM-receptor interaction, which has been associated with spinal disc degeneration[57] and neuropathic pain in a rodent spared nerve injury model[58]. In the chronic T1 versus acute group comparison, there was significant enrichment for MHC class I and II genes in the antigen processing and presentation pathway, to which the HLA genes belong. A string analysis of the co-expression of genes revealed several clusters of genes. We followed up on this finding by examining the expression levels of genes in our study that are included in those clusters. We found that the chronic T1 and T5 groups had higher expression levels of *OPRM1*, *EGFR*, *OXT*, *GRIN2B*, and *AVP* compared to the acute and normal control groups. The *OPRM1* gene, which codes for the mu opioid 1 receptor[59–61], *EGFR* interactions with epiregulin[62], *OXT*, which codes for oxytocin[63–66], and *GRIN2B*, which codes for the N-methyl-D-aspartate (NMDA) glutamate receptor 2b subunit[67–69] are all known to be significant components in pain processing.

We also found, in the acute versus chronic T1, a significant cluster of genes (*MT-ND1*, *MT-ND2*, *MT-ND5*, *MT-ND6*, and *MT-CO3*) centered around *MT-ND2*, the gene encoding for mitochondrial encoded NADH dehydrogenase 2. Single nucleotide polymorphisms (SNPs) in the *MT-ND2* gene have been shown to be associated with oxidative phosphorylation system deficiency, which causes a variety of inborn errors of energy metabolism depending upon the specific SNPs mutated and the affected gene(s)[70]. SNPs in other MT-associated genes have been shown to be associated with migraine headache[71], a pain disorder that is very common in patients with maternally inherited mitochondrial dysfunction[72]. In the chronic T1 versus acute group, all of the mitochondrial associated genes were significantly downregulated. These genes were not found to be differentially regulated in the chronic T5 patients compared with healthy controls, nor did this gene cluster appear in the gene co-expression analysis of the cLBP cohort versus healthy controls. Mitochondria have a critical role in cellular energy metabolism and produce nearly all of the cellular energy via enzymatic coupling of oxidative phosphorylation[73]. One of the most immediate consequences of a deficiency in the oxidative phosphorylation system is an increase the production of superoxide, which can lead to increased production of reactive oxygen species (ROS)[74–76]. Oxidative stress has been shown to be a contributor of a variety of chronic pain conditions including those with inflammatory and neuropathic components[77–79]. Thus, our results suggest that in addition to known pain and MHC locus genes, (including upregulation of *HLA-DMA* and mitochondrial gene, *PDF)*, that blood gene expression of mitochondrial genes involved in oxidative phosphorylation could be potentially useful as biomarkers of those at risk for developing cLBP at a very early time point.

This paper has several limitations that warrant caution in interpreting the results. First, due to the design of the study we were limited to analyzing baseline data and samples of the acute group because data was not collected after pain resolution. In addition, due to the small sample sizes, we did not examine differential gene expression between patients based on sex or race. The second limitation is the lack of congruence between the RNA seq results and several of the genes that we attempted to validate via qPCR. In some respects, one could argue that qPCR validation of RNA seq is a “low tech” method to validate a high-tech method, however there is still interest in doing so. We optimized the primer pairs on non-patient samples and then ran qPCR for the selected genes. One possibility for the lack of congruence in some cases might be that we were amplifying a different isoform of the gene than was captured in the LFC data at the whole gene level. We plan future replication studies with additional samples, which should provide insight into this issue and also, with increased statistical power, provide additional confidence in our findings.

Despite these limitations, chronic low back pain continues to be a leading cause of disability and one of the most expensive medical conditions in the United States[78–80], with nearly 40% of cases of acute low back pain persisting for more than 12 weeks from the time of onset[81–83]. Studies have focused on identifying psychosocial, environmental, structural, and somatosensory factors that might predict who is at risk for developing cLBP[84–88]. However, despite recent findings, the mechanisms underlying the transition from acute to chronic LBP and predictors of those patients who may be at risk of developing chronic pain remain unclear. Thus, we assert that in spite of the limitations, this study is quite innovative, and adds significantly to our body of knowledge regarding potential blood biomarkers of chronic pain. Taken together, the findings from this study identified several genes and pathways that are upregulated at the time of initial onset of pain in patients who develop cLBP compared to patients who have acute resolution of pain and healthy controls, including a set of known pain genes, MHC locus genes, and mitochondrial genes linked to oxidative phosphorylation. This suggests that these genes could be potential blood biomarkers of LBP patients who are at high risk for developing cLBP. The pathways identified offer potential targets for developing new therapeutic strategies for preventing the development of cLBP, or once it forms, to potentially reduce chronic pain in this patient population.

## Supporting information

S1 Table Expanded demographic data. P-values were calculated from ANOVA or Chi-square test(s), whenever applicable.
(DOCX)

S2 Table Differentially expressed genes between chronic T5 group and pain-free normal controls. The DEG set that differentiated chronic T5 patients from healthy normal controls was significantly enriched for known pain genes.
(TXT)

S3 Table Primer sets used for confirmation of expression levels. Primer sets listed were created by the Institute for Genome Sciences, University of Maryland, Baltimore.
(DOCX)

S1 Fig Comparison of expression levels between RNA-sequencing and qPCR. Upregulated expression of the *HLA-A*, *HLADRB5*, *PDF* genes from RNA seq and qPCR were consistent.
(TIFF)
