# Supplementary material for: Whole blood transcriptomic profiles can differentiate vulnerability to chronic low back pain
Source: PLoS One. 2019 May 16;14(5):e0216539. doi: 10.1371/journal.pone.0216539 (PMC6522025; doi:10.1371/journal.pone.0216539)
Supplement: S1 Table — P-values were calculated from ANOVA or Chi-square test(s), whenever applicable. (DOCX) [file pone.0216539.s001.docx]

Table 1. Demographics and Pain Phenotype

|  | **Normal**  **N=21** | **Acute**  **N=11** | **Chronic T1**  **N=13** | **Chronic T5**  **N=19** | **P-value** |
| --- | --- | --- | --- | --- | --- |
| **Age, mean (SD)** | 30.9 (13.9) | 34.4 (9.1) | 38.5 (8.4) | 38.7 (9.0) | 0.184 |
| **Gender** |  |  |  |  | 0.993 |
| **Male, n (%)** | 9 (42.9) | 5 (45.5) | 6 (46.2) | 9 (47.4) |  |
| **Female, n (%)** | 12 (57.1) | 6 (54.4) | 7 (53.8) | 10 (52.6) |  |
| **Race** |  |  |  |  | <0.001 |
| **White, n (%)** | 12 (57.1) | 6 (56.0) | 6 (46.2) | 8 (42.1) |  |
| **Black, n (%)** | 1 (4.8) | 3 (27.0) | 7 (53.8) | 11 (57.9) |  |
| **Other, n (%)** | 8 (38.1) | 2 (18.0) | 0 (0.0) | 0 (0.0) |  |
| **Pain score right now (0-10), mean (SD)** | 0 (0.0) | 2.9 (0.9) | 5.5 (2.5) | 5.7 (2.8) | <0.001 |
| **Pain average score (0-10), mean (SD)** | 0 (0.0) | 3.4 (1.6) | 5.2 (2.0) | 5.1 (2.4) | <0.001 |
| **Heat (warm) threshold, mean (SD)** | 35.3 (1.1) | 35.2 (0.8) | 36.5 (2.5) | 36.4 (2.2) | 0.091 |
| **Heat pain (medium), mean (SD)** | 42.3 (2.2) | 39.4 (4.3) | 40.1 (2.9) | 39.9 (3.1) | 0.034 |
| **Heat pain tolerance, mean (SD)** | 43.3 (3.7) | 40.5 (4.6) | 40.0 (3.0) | 39.5 (3.3) | 0.008 |
| **Pain medication (opioid), n(%)** | 0 (0.0) | 0 (0.0) | 0 (0.0) | 0 (0.0) | -- |
| **Pain medication (NSAID/Tylenol), n(%)** | 0 (0.0) | 7 (63.6) | 3(23.1) | 6(31.6) | <0.001 |
| ^a^p-values were calculated from ANOVA or Chi-square test (Fisher’s exact test), whenever applicable | | | | | |

|  | Overall  N=40 | Normal  N=21 | Chronic T5  N=19 | P-value |
| --- | --- | --- | --- | --- |
| **Age, mean(SD)** | 34.92 (12.01) | 31.48 (13.48) | 38.74 (9.02) | 0.051 |
| **BMI, mean(SD)** | 27.27 (5.7) | 25.91 (4.87) | 28.76 (6.28) | 0.122 |
| **Exercise Days, mean(SD)** | 3.02 (2.01) | 2.95 (1.83) | 3.11 (2.23) | 0.815 |
| **Hours Sleep, mean(SD)** | 6.86 (1.23) | 6.93 (1.16) | 6.79 (1.33) | 0.728 |
| **Any medication use, n(%)** |  |  |  | 0.709 |
| **No** | 30 (75.0) | 15 (71.0) | 15 (79) |  |
| **Yes** | 10 (25.0) | 6 (29.0) | 4 (21) |  |
| **Sex, n(%)** |  |  |  | 0.613 |
| **Male** | 18 (45.0) | 9 (42.9) | 9 (47.4) |  |
| **Female** | 22 (55.0) | 12 (57.1) | 10 (52.6) |  |
| **Race, n(%)** |  |  |  | 0.182 |
| **White** | 20 (50.0) | 12 (57.1) | 8 (42.1) |  |
| **AA** | 12 (30.0) | 1 (4.8) | 11 (57.9) |  |
| **Other** | 8 (20.0) | 8 (38.1) | 0 (0.0) |  |
| **Marital status, n(%)** |  |  |  | 0.607 |
| **Married** | 7 (17.5) | 4 (19.0) | 3 (16.0) |  |
| **Not married** | 33 (82.5) | 17 (81.0) | 16 (84.0) |  |
| **Income, n(%)** |  |  |  | 0.481 |
| **Less than 60K** | 23 (57.5) | 12 (45.5) | 11 (58.0) |  |
| **>=60K** | 17 (42.5) | 9 (54.5) | 8 (42.0) |  |
| **Education, n(%)** |  |  |  | 0.002 |
| **High school** | 7 (17.5) | 0 (0.0) | 7 (37.0) |  |
| **Some college or above** | 33 (92.5) | 21 (100.0) | 12 (63.0) |  |
| **Employment, n(%)** |  |  |  | 0.002 |
| **Not full time** | 12 (33.3) | 2 (9.5) | 10 (52.6) |  |
| **Full time employed** | 28 (66.7) | 19 (90.5) | 9 (47.4) |  |
| **Smoking, n(%)** |  |  |  | 0.114 |
| **No** | 28 (70.0) | 17 (80.9) | 11 (57.9) |  |
| **Yes** | 12 (30.0) | 4 (19.1) | 8 (42.1) |  |
| **Alcohol drinking, n(%)** |  |  |  | 0.977 |
| **No** | 9 (22.5) | 2 (45.5) | 7 (36.8) |  |
| **Yes** | 31 (77.5) | 19 (54.5) | 12 (63.2) |  |
| ^a^p-values were calculated from *t*-tests or Chi-square test (Fisher’s exact test), whenever applicable | | | | |

Table 2. Demographics of chronic T5 vs. pain-free (normal) participants

Table 3. Demographics of chronic T1 vs. acute participants

|  | **Overall**  **N=24** | **Acute**  **N=11** | **Chronic T1**  **N=13** | **P-value** |
| --- | --- | --- | --- | --- |
| **Age, mean(SD)** | 36.58 (8.76) | 34.4 (9.1) | 38.46 (8.38) | 0.267 |
| **BMI, mean(SD)** | 27.54 (5.38) | 26.85 (5.41) | 28.11 (5.5) | 0.579 |
| **Exercise Days, mean(SD)** | 3.42 (2.17) | 2.64 (2.25) | 4.08 (1.93) | 0.111 |
| **Hours Sleep, mean(SD)** | 6.42 (1.32) | 6.18 (1.47) | 6.62 (1.21) | 0.445 |
| **Any medication use, n(%)** |  |  |  | 0.649 |
| **No** | 18 (75) | 9 (81.80) | 9 (69.2) |  |
| **Yes** | 6 (25) | 2 (18.2) | 4 (30.8) |  |
| **Sex, n(%)** |  |  |  | 0.973 |
| **Male** | 11 (45.8) | 5 (45.5) | 6 (46.2) |  |
| **Female** | 13 (54.2) | 6 (54.5) | 7 (53.8) |  |
| **Race, n(%)** |  |  |  | 0.182 |
| **White** | 12 (50) | 6 (54.5) | 6 (16.2) |  |
| **AA** | 10 (41.7) | 3 (27.3) | 7 (53.8) |  |
| **Other** | 2 (8.3) | 2 (18.2) | 0 (0) |  |
| **Marital status, n(%)** |  |  |  | 0.891 |
| **Married** | 8 (33.3) | 4 (36.4) | 4 (30.8) |  |
| **Not married** | 16 (66.7) | 7 (63.6) | 9 (69.2) |  |
| **Income, n(%)** |  |  |  | 0.375 |
| **Less than 60K** | 15 (62.5) | 6 (45.5) | 9 (30.8) |  |
| **>=60K** | 9 (37.5) | 5 (54.5) | 4 (69.2) |  |
| **Education, n(%)** |  |  |  | 0.027 |
| **High school** | 8 (33.3) | 1 (90.9) | 7 (16.2) |  |
| **Some college or above** | 16 (66.7) | 10 (9.1) | 6 (53.8) |  |
| **Employment, n(%)** |  |  |  | 0.033 |
| **Not full time** | 8 (33.3) | 1 (9.1) | 7 ( 53.9) |  |
| **Full time employed** | 16 (66.7) | 10 (90.9) | 6 (46.2) |  |
| **Smoking, n(%)** |  |  |  | 0.240 |
| **No** | 14 (58.3) | 8 (72.7) | 6 (46.2) |  |
| **Yes** | 10 (41.7) | 3 (27.3) | 7 (53.8) |  |
| **Alcohol drinking, n(%)** |  |  |  | 0.729 |
| **No** | 10 (41.7) | 5 (45.5) | 5 (38.5) |  |
| **Yes** | 14 (58.3) | 6 (54.5) | 8 (61.5) |  |
| ^a^p-values were calculated from *t*-tests or Chi-square test (Fisher’s exact test), whenever applicable | | | | |
