# Supplementary material for: Whole blood transcriptomic profiles can differentiate vulnerability to chronic low back pain
Source: PLoS One. 2019 May 16;14(5):e0216539. doi: 10.1371/journal.pone.0216539 (PMC6522025; doi:10.1371/journal.pone.0216539)
Supplement: S3 Table — Primer sets listed were created by the Institute for Genome Sciences, University of Maryland, Baltimore. (DOCX) [file pone.0216539.s003.docx]

|  | Forward-Reverse |  |
| --- | --- | --- |
| ARL2BP | GACGCCTTAGAAGGAGAGAGC | CTCCAGGTAGTACTTGTCCATGA |
| CXCL8 | CTCTTGGCAGCCTTCCTGAT | TGGGGTGGAAAGGTTTGGAG |
| GAPDH | GCTCATTTCCTGGTATGACAACG | GAGATTCAGTGTGGTGGGGG |
| HLA-A | CAGTCACAGACTGACCGAGT | ACGTCGCAGCCATACATTATCT |
| HLA-C | GTGCCATATGCAGCACGAGG | TCCAAGGACAGCTAGGACAAC |
| HLA-DRB5 | GAGGTTCCTACATGGCAAAGC | CCGTCCCGTTGAAGAAATGACAC |
| LILRA2 | GTGGGGACCTACAGATGCTA | ATGTAGTCGTGGAGTCTGTCT |
| PDF | CCTGCGCGTGTTCGTGA | GTTCTCCATTGGGGTCCAGC |
| RNCR3 | AGTCGTTGGGCTATGTGGAC | GCGTGAGGTGCTCCTTAGAG |
| SEMA6B | GGTGTATGAAGAACTGTATCGGC | GTCCTGTGCAGTCCCCTAAG |
| SRXN1 | CCTCGTGGACACGATCCG | CAGCCCCCAAAGGAGTAGAA |
| WHAMMPL2 | ATGGGTCAACCAAGAACGTCA | AGCGGTCTTCATTTATTTCCCC |

Supplemental Table 3. Primer sets used to assess replication of RNA seq results

Following Trizol purification, RNA was processed and sequenced as above. Following mRNA sequencing, eleven DEGs were selected for analysis by real time RT-PCR using GAPDH as an endogenous reference.  For each sample 1 ug of RNA was reverse transcribed to yield cDNA using the SuperScript II First-Strand Synthesis System (Life Technologies). The reverse transcription reactions were then diluted 1:6. A final reaction volume of 20 μL contained 10 μL SYBR Green PCR Master Mix (Roche, Basel, Switzerland), 2μL 2.5μM primers (IDT Technologies, Coralville, Iowa), and 5μL cDNA. For each qPCR, cycling conditions were 50 °C for 2 min, 95 °C for 5 min, 35 cycles of 95 °C for 10 s and 58 °C for 20 s, 72 °C for 65 s using a Roche lightcycler 480 II (Roche, Basel, Switzerland).
