# Supplementary figures and images for: Whole blood transcriptomic profiles can differentiate vulnerability to chronic low back pain
Source: PLoS One. 2019 May 16;14(5):e0216539. doi: 10.1371/journal.pone.0216539 (PMC6522025; doi:10.1371/journal.pone.0216539)

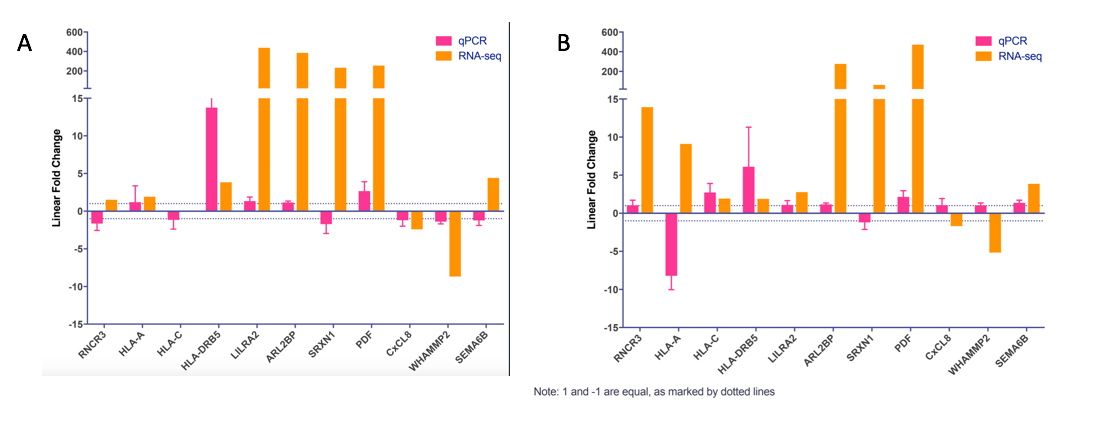

Supplement: S1 Fig — Upregulated expression of the HLA-A, HLADRB5, PDF genes from RNA seq and qPCR were consistent. (TIFF) [file pone.0216539.s004.tiff]
